# Critical Tetramine Poisoning after Sea Snail Ingestion in a Patient on Peritoneal Dialysis: A Case Report

**DOI:** 10.3390/medicina57060564

**Published:** 2021-06-02

**Authors:** In-Hwan Yeo, Jeong-Hoon Lim

**Affiliations:** 1 Department of Emergency Medicine, School of Medicine, Kyungpook National University, Daegu 41944, Korea; inani1113@gmail.com; 2 Division of Nephrology, Department of Internal Medicine, School of Medicine, Kyungpook National University, Daegu 41944, Korea

**Keywords:** tetramine, end-stage kidney disease, peritoneal dialysis, respiratory failure, sea snail

## Abstract

Tetramine in gastropods can cause poisoning symptoms with various side effects. Most of these symptoms are mild and spontaneously resolved due to the rapid excretion of tetramine through the kidneys; however, patients with kidney dysfunction can present severe symptoms. A 48-year-old woman with end-stage kidney disease due to diabetic nephropathy and undergoing peritoneal dialysis (PD) visited our emergency department (ED) with complaints of general weakness, vomiting, and shortness of breath after ingesting some sea snails. On ED arrival, she was in a respiratory failure state; therefore, invasive mechanical ventilation was immediately initiated. Chest radiography showed diffuse severe pulmonary edema and her vital signs fluctuated; thus, continuous renal replacement therapy (CRRT) was initiated at the intensive care unit to treat tetramine intoxication and control volume status. Her condition gradually improved, and she was successfully weaned from mechanical ventilation on the 5th day of admission and moved to the general ward on the 10th day. CRRT was switched to PD. She fully recovered and was discharged on the 15th day of admission. Therefore, clinicians should explain the risk associated with gastropod ingestion to patients with kidney dysfunction and recognize that the clinical course of tetramine toxicity can be critical.

## 1. Introduction

Sea snails, a type of gastropods, are a readily available and edible food in Asian countries such as South Korea and Japan. However, they contain tetramine, which can cause various toxic effects; hence, they should be eaten cautiously [1]. Most toxic effects of tetramine are mild, such as nausea, vomiting, diplopia, and dizziness [2], which spontaneously disappear within a few hours due to its rapid kidney excretion and short half-life [1,3]; however, severe symptoms such as respiratory muscle paralysis can occur in rare cases [4]. In particular, due to renal tetramine excretion, patients with kidney dysfunction may present severe symptoms in tetramine poisoning [5].

In this case report, we present a patient with end-stage kidney disease (ESKD) undergoing peritoneal dialysis (PD), who experienced critical tetramine poisoning requiring invasive mechanical ventilation and continuous renal replacement therapy (CRRT) during hospitalization.

## 2. Case Presentation

**Figure 1:**
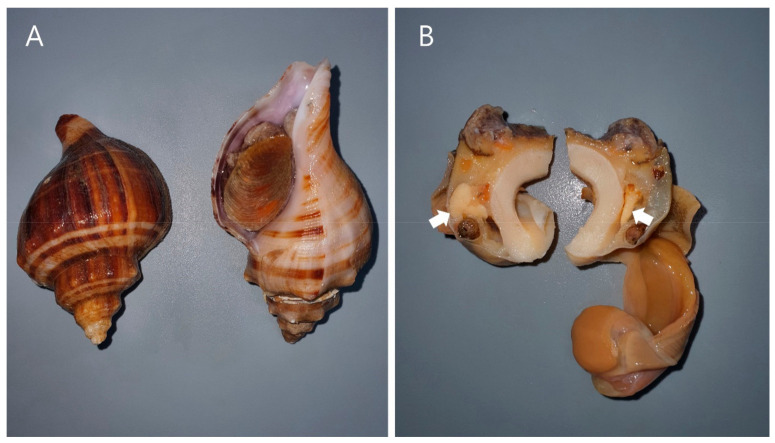
*Neptunea cumingii*, one of the gastropods that contains tetramine (**A**) and the salivary glands (white arrows) that contain the most tetramine (**B**).

The patient was a 48-year-old woman with ESKD caused by diabetic nephropathy and undergoing PD for 10 months. She was on continuous cycling PD and dialyzed 7 days/week with 1.5% dextrose solution (2 L of dwell volume) for 9 h with five exchanges for a night and a long day dwell with 2 L of icodextrin; her residual urine volume was 200 mL/day. Her condition had been stable since the PD initiation without edema, but she suddenly visited to our emergency department with complaints of general weakness, nausea, vomiting, and shortness of breath after sea snail (Figure 1A) ingestion on 4th December 2020. About 3 h before the hospital visit, she ingested 7 boiled sea snails without salivary gland (Figure 1B) removal. There was no evidence of alternate source of poisoning except sea snails. Approximately 30 min after ingestion, dizziness, blurred vision, abdominal pain, and diarrhea occurred. Five of the seven family members who ingested sea snails also felt similar symptoms. At 90 min after ingestion, she developed general weakness, nausea, vomiting, and shortness of breath.

**Figure 2:**
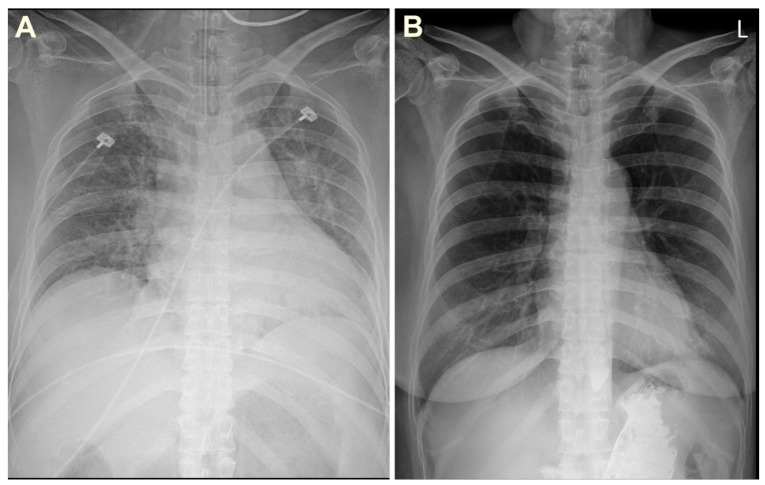
Chest radiographs on admission (**A**) and discharge (**B**).

In the emergency department, her initial blood pressure was 112/79 mmHg; pulse rate, 107 beats/min; respiratory rate, 20 breaths/min; axillary temperature, 36.0 °C; and peripheral oxygen saturation, 50% measured by a pulse oximeter. Chest auscultation revealed crackles in both lungs. The initial white blood cell count was 11.17 × 10^3^/μL with 58.1% neutrophils and 0.5% eosinophils. The results of arterial blood gas analysis were: pH, 7.010; PaCO_2_, 84.8 mmHg; PaO_2_, 61.4 mmHg; and HCO_3_¯, 15.4 mmol/L. The serum glucose concentration was within normal range (130 mg/dL); the sodium level was normal (138 mmol/L); the potassium level was elevated (5.6 mmol/L); the calcium level was normal (8.9 mg/dL); the aspartate transaminase and alanine transaminase levels were normal (32 U/L and 17 U/L); the ammonia level was normal (32 μmol/L); and the lactic acid level was normal (1.5 mmol/L). The patient did not have acute inflammatory signs such as being febrile or chilly, and the high-sensitivity C-reactive protein level was also within normal range (0.24 mg/dL). No evidence of peritonitis was observed in the peritoneal fluid analysis. The initial chest radiograph showed diffuse severe pulmonary edema with peribronchial infiltration and subsegmental atelectasis (Figure 2A).

**Figure 3:**
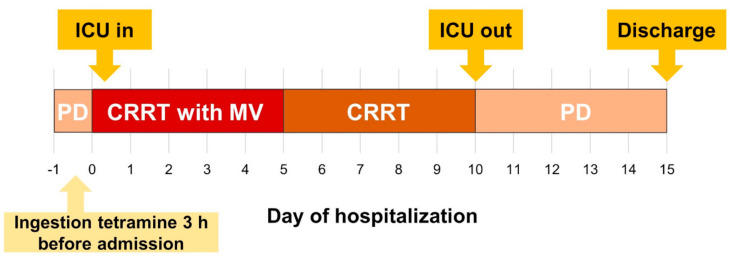
Clinical course of the patient. Abbreviations: ICU, intensive care unit; PD, peritoneal dialysis; CRRT, continuous renal replacement therapy; MV, mechanical ventilation.

Upon emergency department arrival, her mental status was drowsy and she was in a state of respiratory failure; therefore, intubation and invasive mechanical ventilation were immediately performed. The patient’s vital signs fluctuated; thus, PD was discontinued, and CRRT was initiated (target clearance dose: 35 mL/kg/h) in the intensive care unit to remove blood tetramine and control volume status (Figure 3). Her symptoms gradually improved, and mechanical ventilation weaning succeeded on the 5th day of admission. CRRT was switched back to PD on the 10th day of admission. She fully recovered without pulmonary edema (Figure 2B) and was discharged on the 15th day after admission.

## 3. Discussion

This case report showed the risk of tetramine intoxication in patients with ESKD on PD. In general, tetramine toxicity causes mild symptoms, which spontaneously disappear within several hours. However, in rare cases especially in patients with kidney diseases, it can cause critical symptoms that require intensive care.

Tetramine, also known as, tetramethylammonium ion, (CH_3_)_4_N^+^, is naturally found in gastropods such as the *Neptunea* species and causes poisoning when ingested in large amounts. Its structural formula is similar to that of acetylcholine (C_7_H_16_O_2_); therefore, its toxicity mechanism is to inhibit nicotinic acetylcholine receptors and act as a ganglionic blocking agent that blocks synaptic transmission [1,6]. Tetramine poisoning is mainly characterized by gastrointestinal symptoms, such as nausea, vomiting, and abdominal pain, and neurologic symptoms, such as headache, dizziness, diplopia, sleepiness, amblyopia, neck stiffness, arm and leg paralysis, and gait disturbance [2,7]. In general, symptoms develop within 30 min after ingestion, and the half-life is 60 min [1,3]. Respiratory failure was rarely reported in humans; only one patient has been reported with respiratory muscle paralysis but spontaneously improved within several hours [4]. However, tetramine poisoning has been confirmed to also cause severe pulmonary edema in addition to respiratory muscle paralysis that requires invasive mechanical ventilation.

The threshold of tetramine concentration that causes toxic symptoms is known to be 10 to 50 mg and the average-weighted *Neptunea* species contain 17.3 mg of tetramine [7,8]. Therefore, the patient would have taken approximately 120 mg of tetramine. The tetramine concentration in gastropods is also known to vary seasonally [6]. In South Korea, the tetramine concentration in gastropods is considerably higher in December than that in April [9]; therefore, special caution should be taken during winter. The tetramine distribution in gastropods has been confirmed in a previous study, showing that the salivary glands contain the highest concentration, followed by the meat and midgut gland [2]. Tetramine is stable against heat and causes toxic effects even when boiled, and toxicity can occur even with the boiled water itself. In particular, when cooked in its shell, tetramine in the salivary gland has been found to spread [2]; therefore, salivary glands should be removed before cooking.

Tetramine has a molecular weight of 74 Da and is mostly excreted by the kidneys (>95%) when a large amount is accumulated [3]. Tetramine is excreted without chemical changes and both glomerular filtration and carrier-mediated secretion are involved for the excretion [3]. In patients with decreased renal function with decreased excretion capacity, toxicity can be severe and last longer than with healthy individuals; therefore, special caution is required. With its low molecular weight, serum tetramine can be sufficiently removed by renal replacement therapy [5]. A case of tetramine toxicity has been reported in patients with ESKD on hemodialysis [5], but not yet in patients on PD. PD can continuously remove uremic toxins, and our patient was on PD during tetramine ingestion. However, it was before the automated PD, so the clearance was low and the toxin would have not been cleared. Although we did not measure the serum concentration of tetramine, the patient’s initial symptoms and medical history were consistent with tetramine poisoning. In addition, immunological tests such as mast cell tryptase level were not performed, but the patient’s symptoms were different from typical allergic reactions such as anaphylaxis. Therefore, we confirm that in patients on PD, tetramine poisoning can occur and can be serious.

In conclusion, clinicians should emphasize the risk associated with gastropod ingestion to patients with kidney dysfunction and recognize that the clinical course of tetramine toxicity in patients with ESKD can be critical.

## Data Availability

Not applicable.
